# Distribution of selected healthcare resources for influenza pandemic response in Cambodia

**DOI:** 10.1186/1475-9276-12-82

**Published:** 2013-10-04

**Authors:** Sara U Schwanke Khilji, James W Rudge, Tom Drake, Irwin Chavez, Khieu Borin, Sok Touch, Richard Coker

**Affiliations:** 1 Communicable Diseases Policy Research Group, London School of Hygiene & Tropical Medicine, 9th Floor, Satharanasukwisit Building, Mahidol University, 420/1 Rajavithi Road, Bangkok 10400, Thailand; 2 Faculty of Tropical Medicine, Mahidol University, Bangkok 10400, Thailand; 3 Centre for Livestock & Agriculture Development (CelAgrid), Phnom Penh, Cambodia; 4 Department of Communicable Disease Control, Cambodia Ministry of Health, 151-153 Kampuchea Ground Avenue, Phnom Penh, Cambodia

**Keywords:** Health system, Pandemic influenza, Resource mapping, Resource, Allocation, Antivirals, Cambodia, Southeast Asia

## Abstract

**Introduction:**

Human influenza infection poses a serious public health threat in Cambodia, a country at risk for the emergence and spread of novel influenza viruses with pandemic potential. Prior pandemics demonstrated the adverse impact of influenza on poor communities in developing countries. Investigation of healthcare resource distribution can inform decisions regarding resource mobilization and investment for pandemic mitigation.

**Methods:**

A health facility survey performed across Cambodia obtained data on availability of healthcare resources important for pandemic influenza response. Focusing on five key resources considered most necessary for treating severe influenza (inpatient beds, doctors, nurses, oseltamivir, and ventilators), resource distributions were analyzed at the Operational District (OD) and Province levels, refining data analysis from earlier studies. Resources were stratified by respondent type (hospital vs. District Health Office [DHO]). A summary index of distribution inequality was calculated using the Gini coefficient. Indices for local spatial autocorrelation were measured at the OD level using geographical information system (GIS) analysis. Finally, a potential link between socioeconomic status and resource distribution was explored by mapping resource densities against poverty rates.

**Results:**

Gini coefficient calculation revealed variable inequality in distribution of the five key resources at the Province and OD levels. A greater percentage of the population resides in areas of relative under-supply (28.5%) than over-supply (21.3%). Areas with more resources per capita showed significant clustering in central Cambodia while areas with fewer resources clustered in the northern and western provinces. Hospital-based inpatient beds, doctors, and nurses were most heavily concentrated in areas of the country with the lowest poverty rates; however, beds and nurses in Non-Hospital Medical Facilities (NHMF) showed increasing concentrations at higher levels of poverty.

**Conclusions:**

There is considerable heterogeneity in healthcare resource distribution across Cambodia. Distribution mapping at the local level can inform policy decisions on where to stockpile resources in advance of and for reallocation in the event of a pandemic. These findings will be useful in determining future health resource investment, both for pandemic preparedness and for general health system strengthening, and provide a foundation for future analyses of equity in health services provision for pandemic mitigation planning in Cambodia.

## Introduction

The serious public health impact of human influenza infection in Cambodia is increasingly apparent. Highly pathogenic avian influenza is now considered enzootic among poultry in the country, with a total of 34 human cases of H5N1 infection confirmed as of early July 2013, of which 28 have been fatal [1,2]. At 82%, the case fatality rate for H5N1 in Cambodia is second only to Indonesia’s (83%) in the list of countries with sustained cases [2]. Situated within Southeast Asia, a region recognized as playing a central role in the circulation and evolution of influenza viruses [3,4], Cambodia is at risk for the emergence and spread of novel strains with pandemic potential. Strategies to contain and mitigate future influenza pandemics in low- and middle-income countries are critically important, both because many of these countries, like Cambodia, are in regions with very high risk of emerging infections and because health systems in these areas are weaker compared to those in high-income countries [5-7]. Developing countries are likely to be at particular risk in the event of future influenza pandemics, with one study predicting that they will bear the burden of more than 90% of influenza-related deaths globally [8]. Within developing countries, too, poor populations are disproportionately affected by the health impacts of pandemics, in part due to decreased access to efficiently functioning healthcare systems; this vulnerability was illustrated in the higher rates of infection and greater morbidity and mortality among poor communities during the H1N1 pandemic of 2009 [9].

The H1N1-2009 pandemic also demonstrated the limitations of health system pandemic response capacities, even in developed countries [10,11]. While much emphasis has been placed on strengthening surveillance and outbreak response activities throughout South East Asia and globally, there is increasing recognition of the need to employ resource mobilization and redistribution strategies in pandemic mitigation planning (that is, activities aimed to reduce morbidity and mortality in the event of a pandemic) [12] and to evaluate critically existing and future investments in heath system resources. This has proven true in Cambodia, where semi-structured interviews conducted with members of the pandemic influenza national coordinating committees, performed in response to the 2009 H1N1 swine flu pandemic, revealed a need for data to inform health system investment allocation for maximum efficiency in pandemic mitigation strategies [13]; the research described here was performed in response to this identified need. Although resource-constrained and largely dependent on external donors for health sector financing, Cambodia has proven its ability to successfully increase investment in health care in the recent past, with an explicit goal of achieving more efficient and equitable distribution of health care resources. The country has made remarkable achievements in rebuilding its national health system since its systematic destruction under the Khmer Rouge regime in the 1970s, including the introduction of health system reforms in the 1990s focused on delivering primary health care nationwide through a district-based system. Since the development of this system, important advancements have been made in the health status of Cambodians, including improvements in life expectancy and infant mortality [14]. The country is now divided into 77 Operational Districts (ODs) on the basis of population density (100,000 – 200,000 people per OD), each supporting a network of referral hospitals, health centers and health posts that provide basic health care at the local level [15]. In order to strengthen pandemic influenza mitigation in Cambodia, a clearer understanding of current healthcare resource distribution at the OD (as opposed to Province) level is required.

This report builds upon the findings of a previous research project, AsiaFluCap [16]. The AsiaFluCap Project analyzed how health system resources can be most effectively and efficiently deployed across selected Asian countries (Cambodia, Indonesia, Lao PDR, Taiwan, Thailand and Vietnam) in the event of a pandemic. Using a mathematical transmission model to simulate a mild to moderate pandemic influenza scenario, gaps in health care resources key for responding to pandemic influenza were estimated within and compared across countries, with calculation of potential mortality burden associated with those gaps. The results of these analyses, carried out at the Province level in all study countries, revealed high variability in resource capacities both between and within countries, with substantial avoidable mortality attributed to severe shortages in mechanical ventilators [12]. A follow-up analysis, focusing only on study countries in the Greater Mekong Subregion (Cambodia, Lao PDR, Thailand and Vietnam) mapped and analyzed key health care resource distributions at the Province level in order to identify relative resource shortages, mismatches, clustering, and inequalities in distribution [17]. Again, a high degree of heterogeneity in resource distribution was noted. In Cambodia, the availability of key resources was found to be particularly low and unequally distributed compared to the other study countries, suggesting that a pandemic influenza here could have a disproportionately high public health impact.

The current analysis constitutes a sub-component of an overarching project, the CamFlu Project, which draws on current health service resource distribution patterns in Cambodia to model scenarios for cost-effective options to improve pandemic response capacity and evaluate the public health impact of scaling up resources for influenza pandemic response. Thus, the present study aims to further explore the challenges involved in efficiently and effectively distributing key health system resources in Cambodia for pandemic influenza mitigation, specifically by refining previous distribution analyses from the Province to the OD level and investigating associations with a socioeconomic indicator, the Predicted Family Poverty Rate (PPR).

## Methods

### Data collection on key resources

This analysis focuses on five key health system resources, selected due to their importance for responding effectively to pandemic influenza: inpatient beds, doctors, nurses, oseltamivir, and mechanical ventilators. These resources where chosen from a pool of 57 health resource items, including health care facilities, human resources, medical equipment, medications, laboratory capacity, and communication technology, identified through a two-step process of systematic literature review and Delphi consensus by an expert panel. At a follow-up project consortium workshop attended by health officials, public health and health system researchers, medical doctors, and epidemiologists from across several Asian and European countries, the five key health system resources were selected as being the strongest indicators of healthcare capacity for pandemic mitigation. Data were collected as part of the AsiaFluCap Project, using survey tools as described in Hanvoravongchai *et al*. [17]. In brief, itemized healthcare resources were captured and quantified via questionnaires administered to all government-supported hospitals (n=185) and District Health Offices (DHOs) throughout all ODs (n=77) in Cambodia during March 2009 to September 2009. A map of ODs in Cambodia is given in Additional file 1. Separate questionnaires were designed for and administered to hospital-based and DHO-based respondents. The hospital questionnaire assessed resource availability within a given health facility, whereas the DHO questionnaire elicited information on health resources available in district health facilities (e.g. health centers, health posts) outside the hospital setting, hereafter referred to as Non-Hospital Medical Facilities (NHMF). The district questionnaire emphasized the importance of including data on non-hospital resources only, in an effort to avoid double-counting. Sample photographs and definitions of all health resource items were included at the end of the questionnaires. The survey tools were translated into Khmer and validated by reverse translation. Missing data from the primary survey due to non-responses or incomplete answers were extrapolated through linear prediction models using STATA version 11, as described previously [12].

### Data analysis

Per capita resource densities (per 10,000 population) were calculated at the OD level in addition to the Province level for all key healthcare resources, increasing the spatial resolution of resource densities reported previously in the AsiaFluCap Project [17]. The distribution of resource densities at each of these levels was considered in a number of ways: (i) application of a measure of distributional inequality (the Gini coefficient), (ii) identification of relatively under- and over-supplied areas, (iii) geographical information system (GIS) analysis of resource clustering, and (iv) analysis of resource density by average poverty rates (the PPR), a measure of socioeconomic status not included in prior analyses.

Per capita distribution of the five key resources was first assessed by calculating the Gini coefficient, a commonly used index of relative inequality [18,19]. Gini coefficients for each resource were calculated at the OD and Province levels based on resource densities, both with and without weighting for area population sizes. Corresponding Lorenz curves were also plotted for each resource at both OD and Province levels.

Geographical resource distribution was analysed at the OD and Province levels to identify areas of relative under- and over-supply, according to the following definitions (adapted from the AsiaFluCap Project [17]). **Relatively undersupplied** areas for a given resource are those ODs or Provinces with a resource density within the lowest quintile of all resource densities across ODs or Provinces, respectively, country-wide. Areas with more than one relatively undersupplied resource are considered to have **multiple under-supply** in resource provision. **Relatively oversupplied** areas for a given resource are those ODs or Provinces with a resource density within the highest quintile across the country. Areas with more than one relatively oversupplied resource are designated as having **multiple over-supply** provision of resources. In some ODs and Provinces, areas of **resource mismatch**, defined as regions relatively undersupplied for one or more resources and relatively oversupplied for at least one other resource, were identified. It is important to note that the designations of relative under- and oversupply are, by definition, comparative and should not be interpreted as connoting *adequacy* of resource availability.

GIS analysis was performed using ESRI® ArcGIS 9.0 and OpenGeoDA 1.0.1 to identify resource **clustering** or **dispersion** across ODs and Provinces. Clustering or dispersion of resources occurs in areas where the density of a given resource significantly correlates with or differs from surrounding areas (spatial autocorrelation). Spatial clustering of areas with similar resource densities (either high or low) is defined by positive spatial autocorrelation, while spatial outliers (where an area of high resource density is surrounded by areas with low resource densities, or vice versa) are defined by negative spatial autocorrelation. Moran’s index for global spatial autocorrelation, which reflects correlation between resource density in a given area and average resource density in neighboring areas [20], was used to identify resource clustering or dispersion in this analysis. The spatial weights were constructed using OpenGeoDa with a first-order rook contiguity structure which only considers areas that share borders as influential neighbours [21]. This type of contiguity was chosen with the assumption that the flow or exchange of the surveyed resources move through authorized means and monitored routes. The level of significance was set at *p*<0.05 and simulation runs to 9,999. Local Indicators of Spatial Autocorrelation (LISA) analysis represents an extension to the Moran’s Index and measures the statistical significance of each OD as a cluster and cluster type. The software also employs a correction for areas with relatively smaller populations. For each resource, all ODs were classified as spatial clusters (low-low or high-high), spatial outliers (high-low or low-high), or not statistically significant.

Finally, a potential link between socioeconomic status and resource distribution was explored by incorporating a poverty measure, the PPR, into the geographical analysis. The PPR is a continuous variable, expressed as a percentage, which captures the portion of total families deemed poor in a given area. Based on a multilevel mixed effect regression model developed by the Cambodian National Committee for Sub-National Democratic Development (NCDD) Monitoring & Evaluation Unit, the PPR utilizes commune-level data from the national Commune Database (CDB) and IDPoor, a project supervised by the Ministry of Planning with technical assistance from the Deutsche Gesellschaft für Internationale Zusammenarbeit (GIZ). Rather than utilizing income data to determine poverty levels, the CDB data relies on proxy indicators for poverty, including household assets (e.g. televisions, bicycles vs. motorbikes, and construction material of dwelling) and variables capturing educational attainment (e.g. adult literacy and the ratio of children aged 6–14 in school) [personal communication with Mr Ny Boret, NCDD, 14 August 2013]. Similarly, the IDPoor data identifies poor households on the basis of a standard questionnaire that utilizes proxy indicators for poverty, with a focus on observable and verifiable assets, as well as social indicators such as household composition, dependency ratios, and school attendance [22]. The PPR used in this analysis was published in the Provincial Data Book of 2009 [23].

In order to link the PPR to the resource data obtained through the survey collection described above, a composite PPR was calculated for each OD and Province by determining a weighted average for all communes within the relevant area. Both resource densities and weighted PPR averages were calculated using population data derived from the General Population Census of Cambodia (2008) [24]. All resource densities and weighted PPR averages were calculated as quintiles for comparison. In order to better understand location and accessibility of key resources, resource densities were further broken down by survey respondent type (i.e. hospital or NHMF) prior to analysis. Of note, 2009 reporting of the PPR for communes in Phnom Penh Province was minimal, so weighted PPR averages could not be accurately calculated for this Province or the ODs it contains (Cheung, Tbong, Lech, and Kandal); these ODs were excluded from the analysis. On the basis of the available data, however, it was determined that Phnom Penh Province can reasonably be assumed to fall into the first (i.e. lowest) poverty quintile. Simple linear regression was performed to evaluate for potential interaction between PPR and resource densities (Microsoft Office Excel 2007), and resource densities were mapped against PPR at the OD and Province levels using Arc GIS 10.

## Results

### Survey response

The questionnaire response rates for both government-based hospitals and DHOs was 100 percent. There were two instances of missing data among the variables reported in this analysis, both of which were for oseltamivir (one each from a hospital-based and DHO-based respondent, representing 0.5 percent and 1.3 percent of responses, respectively). Results presented here include extrapolated data for these missing questionnaire items, as described previously [17].

### Statistical distributions

**Figure 1:**
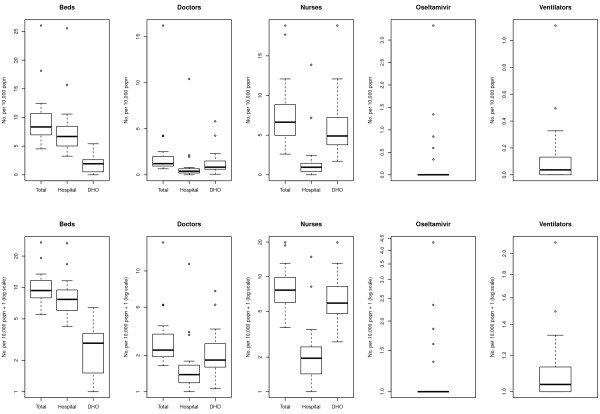
**Availability per 10,000 population of key healthcare resources for responding to pandemic influenza across Provinces in Cambodia.** Boxes represent the interquartile range with the median denoted by the heavy line. Whiskers represent upper and lower outliers within 1.5 times the interquartile range.

**Figure 2:**
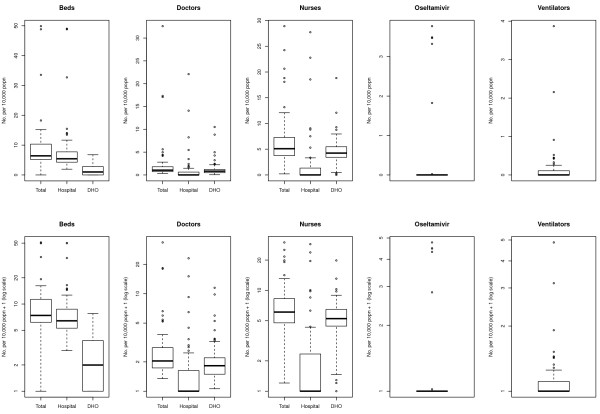
**Availability per 10,000 population of key healthcare resources for responding to pandemic influenza across Operational Districts in Cambodia.** Boxes represent the interquartile range with the median denoted by the heavy line. Whiskers represent upper and lower outliers within 1.5 times the interquartile range.

The statistical distributions of the densities (per 10,000 population) for the five key resource items across Provinces and ODs are presented as box plots in Figures 1 and 2, respectively. For each resource, a box plot representing the total number of available resources is presented, as well as a breakdown by location of respondent (hospital vs. DHO); for ease of interpretation, results are plotted on both linear and logarithmic scales. Inpatient beds were generally concentrated within hospitals rather than in NHMF settings; this pattern was amplified at the Province level. Resource density of doctors was generally low across clinical settings (hospital vs. NHMF) and administrative level (OD vs. Province), although there were a number of high outliers, particularly amongst hospital-based doctors. A pattern of higher concentration of nurses in NHMF than in hospitals, particularly at the OD level, was observed. The availability of both oseltamivir stockpiles and ventilators was very low across clinical settings and administrative levels.

### Equality of resource distribution

Gini coefficients for each of the five key resources across Provinces and ODs are shown in Table 1, with corresponding Lorenz curves given in Additional file 2. The results demonstrate inequalities in distribution of all resources at the OD level. Oseltamivir and ventilators showed very high levels of inequality in distribution, in large part due to their scarcity throughout most of the country. On the other hand, the distribution of nurses and beds was relatively equal throughout the country at the Province level.

**Table 1:** Inequality in distribution of key resources across Provinces and Operational Districts within Cambodia

	**Gini coefficient**				
	**Inpatient beds**	**Doctors**	**Nurses**	**Oseltamivir**	**Ventilators**
Operational District	**0.36**	**0.57**	**0.36**	**0.94**	**0.88**
Operational District, weighted	**0.41**	**0.64**	**0.39**	**0.92**	**0.85**
Province	0.22	**0.46**	0.26	**0.88**	**0.72**
Province, weighted	0.29	**0.55**	0.29	**0.76**	**0.70**

Gini coefficients for each of the five key resource items by Province and Operational District, including population-weighted calculations. Resources exhibiting high levels of inequality in distribution (Gini coefficient > 0.35) are in **bold**.

### Resource under- and over-supply

As described in the methods section, areas of relative under-supply are classified as those with resource densities in the lowest quintile of resource distribution across all ODs or Provinces, respectively, while areas of relative over-supply are characterized by resource densities in the highest quintile (see Table 2). As the majority of ODs reported zero quantities of oseltamivir and ventilators, these resources were excluded from this portion of the analysis. A total of 23 ODs (29.9%) were classified as areas of relative under-supply, compared with 18 ODs (23.4%) meeting criteria for relative over-supply. An equal number of ODs met the definitions for areas of multiple under- and over-supply, respectively (12 each, or 15.6%); of note, only one OD (Ponhea Krek, in Kampong Cham Province) was multiply undersupplied for all three resource items, whereas six ODs were multiply oversupplied for beds, doctors, and nurses (Battambang, Battambang Province; Cheung, Phnom Penh Province; Kandal, Phnom Penh Province; Lech, Phnom Penh Province; Siem Reap, Siem Reap Province, and Daun Keo, Takeo Province). In terms of population, 28.5% of residents live in areas of relative under-supply vs. 21.3% in areas of relative over-supply; the corresponding figures for areas of multiple under-supply and over-supply are 14.0% and 21.0%, respectively.

**Table 2:** Operational Districts with relative under- and over-supply of key health resources, including multiple under- and over-supply, with populations

	**Relative under-supply**	**Pop**	**Relative over-supply**	**Pop**	**Multiple under-supply**	**Pop**	**Multiple over-supply**	**Pop**
1	Preah Net Preah	123186	Thmar Puok	116785	Mong Russey	178437	Mongkol Borei	240432
2	Sang Ker	174330	Kampong Cham	265104	Chamkar Leu	156779	Battambang	335525
3	Kroch Chhmar	100606	**Oudong**	116655	Ponhea Krek	215808	Kampot (Kampot)	143344
4	Tbong Khmum	206184	Muk Kam Poul	69359	Prey Chhor	166173	Takhmau	235550
5	Kampong Chhnang	208475	Sre Ambel	59911	Srey Santhor	135469	**Smach Meanchey**	62865
6	Kampon Speu	375080	Kratie	Kratie	Kampong Tralach	155518	Cheung	350522
7	**Oudong**	116655	Sen Monorom	61107	Boribo	114212	Kandal	273899
8	Baray Santuk	237036	**Tbeng Meanchey**	171139	Koh Thom	141470	Lech	501358
9	Stong	133194	Neak Loeung	162620	Saang	150755	Siem Reap	345440
10	Angkor Chey	104069	**Peareang**	158748	Mesang	101217	Steung Treng	100967
11	Ang Snuol	83203	Prey Veng	184632	Preah Sdach	112046	Daun Keo	185522
12	Ksach Kandal	117322	Sampov Meas	265218	Sotr Nikum	245639	Kep	35753
13	Ponhea Leu	88607	Banlung	150466				
14	**Smach Meanchey**	62865	**Kralanh**	97211				
15	Tbong	367779	**Mittapheap**	221396				
16	**Tbeng Meanchey**	171139	Svay Rieng	266321				
17	**Peareang**	158748	Samrong	185819				
18	**Kralanh**	97211	Pailin	70486				
19	Angkor Chum	203998						
20	**Mittapheap**	221396						
21	Romeas Hek	115371						
22	Bati	186038						
23	Prey Kabass	149443						

Areas of relative under-supply are defined as those with any resource densities in the lowest quintile of resource distribution across all ODs, while areas of relative over-supply are characterized by any resource densities in the highest quintile. Areas of multiple under-supply are defined as those with >1 resource density in the lowest quintile of resource distribution across all ODs or Provinces, respectively, while areas of relative over-supply are defined as having >1 resource density in the highest quintile. The Province in which each OD is located is included in brackets. **Bold** names indicate ODs with resource mismatch, where a given OD has at least one resource in both the lowest and highest quintiles.

**Figure 3:**
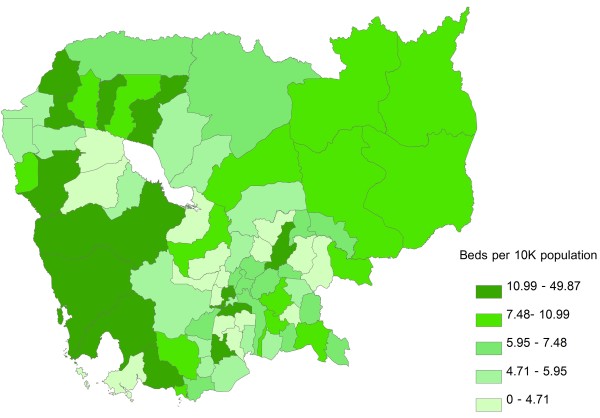
**Geographic distribution of inpatient beds per 10,000 population at the Operational Districts level.** Areas of under- and over-supply are defined as those with the lowest (Quintile 1) and highest (Quintile 5) resource density quintiles, respectively.

**Figure 4:**
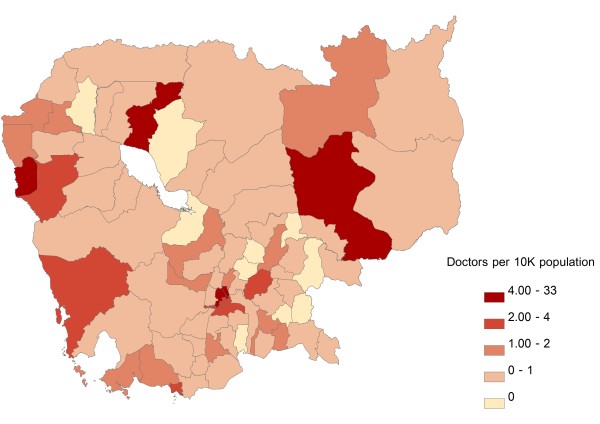
**Geographic distribution of doctors per 10,000 population at the Operational Districts level.** Areas of under- and over-supply are defined as those with the lowest (Quintile 1) and highest (Quintile 5) resource density quintiles, respectively.

**Figure 5:**
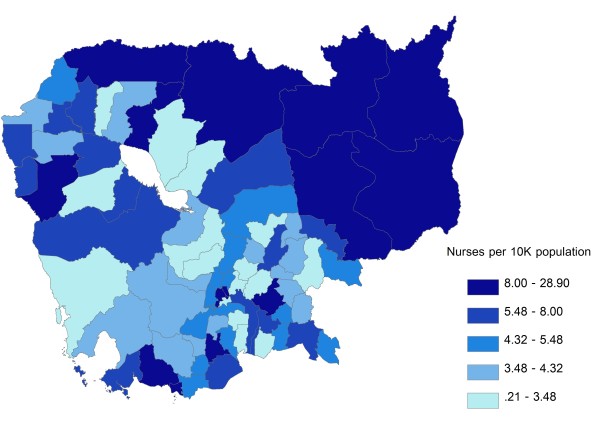
**Geographic distribution of nurses per 10,000 population at the Operational Districts level.** Areas of under- and over-supply are defined as those with the lowest (Quintile 1) and highest (Quintile 5) resource density quintiles, respectively.

Inpatient beds were most heavily concentrated around Phnom Penh Province and in the western parts of the country, with areas of lowest concentration generally clustering in the central parts of the country, including areas surrounding Phnom Penh Province and Tonle Sap (Figure 3). Doctors were likewise heavily concentrated in Phnom Penh Province, with low concentrations around Tonle Sap and in the south-central areas of the country (Figure 4). The geographic distribution of nurses was characterized by a very different pattern, with highest concentration of resources along the north and north-eastern borders of the country; areas of lowest resource concentration were scattered but, similarly to doctors, tended to cluster around Tonle Sap and in the south-central areas of the country (Figure 5). In terms of population affected, 26.8 %, 25.2%, and 26.3% of the Cambodian population live in areas with highest concentrations of inpatient beds, doctors, and nurses, respectively; the corresponding figures for the percentage of population living in areas of lowest resource concentration are 18.5%, 19.9% and 19.7%, respectively. Given the very low absolute numbers of oseltamivir and ventilator supplies throughout the country, and the fact that the great majority of ODs had zero quantities of either supply, GIS distribution figures for these two resources are not included.

### Resource clustering and outliers

Low-low clusters are areas with low resource density surrounded by areas with low resources. Similarly, high-high clusters are areas with high resource per capita surrounded by areas with high per capita resource. High-low and low-high areas are called spatial outliers because of the mismatch those areas exhibit with respect to the resource being considered (areas with high per capita resource surrounded by areas low in resources or areas low in resources surrounded by areas with high per capita resource, respectively).

**Figure 6:**
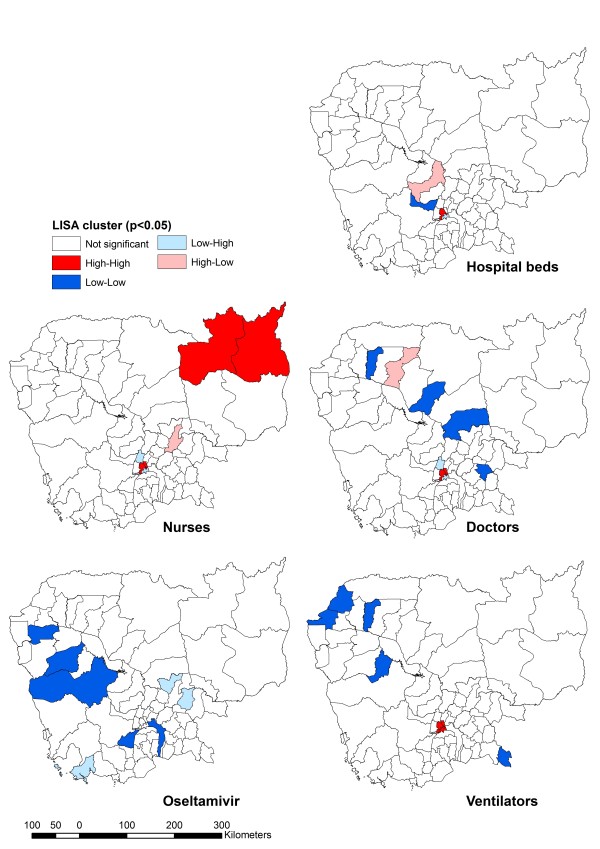
Spatial clustering of key healthcare resources across Provinces in Cambodia (LISA).

With reference to the Moran’s Index (Table 3), all resources except oseltamivir indicated positive spatial autocorrelation but only hospital beds, doctors, and nurses were statistically significant. Among those ODs achieving statistical significance, the adjacent ODs of Cheung, Kandal, and Lech in Phnom Penh were consistently classified as high-high clusters (except for oseltamivir), indicating a concentration of resources in the capital city. The majority of low-low OD clusters lie in the northern and western provinces of Cambodia, suggesting shortages in resources per population, except for nurses (Figure 6). There were few spatial outliers for any resource, and these did not reveal any discernible pattern; the spatial outliers noted, however, are useful for identifying neighboring ODs that can readily share resources when needed.

**Table 3:** Significant LISA cluster/outlier Operational Districts by resource

**Resource**	**Moran’s index; p-value**	**Operational District(s)**			
		**High-high**	**Low-low**	**Low-high**	**High-low**
**Hospital beds**	0.136; 0.040	Kandal, Lech	Oudong	Tbong	Kampong Chhnang
**Doctors**	0.359; 0.004	Cheung, Kandal, Lech	Kralanh, Stong, Baray Santuk, Kamchay Mear	Ponhea Leu, Tbong	Seam Reap
**Nurses**	0.227; 0.008	Banlung, Cheung , Kandal, Lech, Stueng Treng		Ponhea Leu, Tbong	Kampong Cham
**Oseltamivir**	−0.078; 0.058		Kean Svay, Kong Pisey, Mong Russey, Sampov Meas, Thmor Koul	Chamkar Leu, Mittapheap, Tbong Khmum	
**Ventilators**	0.091; 0.056	Cheung, Kandal, Lech, Tbong	Bakan, Chi Phu, Kralanh, Preah Net Preah, Thmar Puok, Uo Chrov		

High-high clusters represent areas with high per capita resource density surrounded by areas with high per capita resource density. Low-low clusters are areas with low per capita resource density surrounded by areas with low per capita resource density. High-low and low-high areas are spatial outliers due to the mismatch these areas exhibit for a given resource (areas with low per capita resource density surrounded by areas with high per capita resource density, and vice-versa).

### Resource distribution by predicted family poverty rate

**Figure 7:**
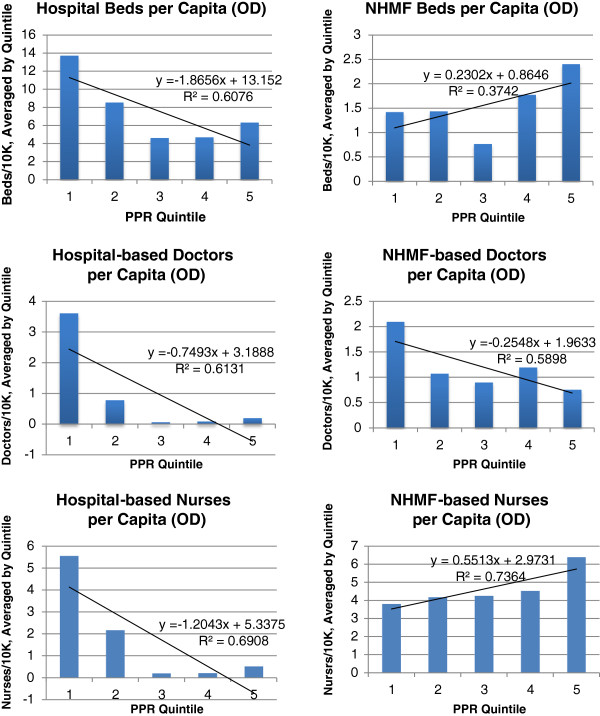
Per capita density of inpatient hospital beds, doctors, and nurses by Predicted Family Poverty Rate, stratified by respondent type (hospital vs. District Health Office) at the Operational District level.

Analysis of resource densities by PPR, stratified by clinical setting (hospital vs. NHMF), revealed discernible patterns (Figure 7). Of note, there was substantial heterogeneity in resource distribution among ODs within a given Province, effectively obscuring patterns when data were analyzed at the Province rather than OD level; thus, results presented here focus only on analysis at the OD level.

The distribution of inpatient hospital beds per capita, plotted against PPR, showed heavy concentration of beds in areas of lowest poverty (first quintile; R^2^ = 0.61), with a U-shaped distribution for the remaining poverty quintiles. Among NHMF, in contrast, there was a nadir in inpatient bed availability in the middle (third) poverty quintile, with resource availability increasing alongside increasing poverty level in the fourth and fifth quintiles (R^2^ = 0.34). Doctors were very heavily concentrated in the wealthiest areas (first quintile), although this pattern was more pronounced in the hospital than NHMF setting (R^2^ = 0.61 and 0.58, respectively). If Phnom Penh OD is excluded from the analysis of NHMF resources, per capita density of doctors is relatively equally distributed across poverty quintiles (see Additional file 3). Hospital-based nurses were distributed similarly to doctors, as they were heavily concentrated in the wealthiest areas (first quintile; R^2^ = 0.69). The distribution of NHMF nurses, however, showed a clear pattern of increasing concentrations in poorer areas (R^2^ = 0.74). Both oseltamivir and ventilators were most heavily concentrated in areas in the first and second poverty quintiles, with supplies sparsely distributed throughout poorer areas (see Additional file 4).

## Discussion

The results presented here reveal notable heterogeneity in distribution of health resources considered critical for pandemic influenza response across Cambodia. This diversity in distribution is related to a number of variables, including resource type (human resources vs. medical supplies), clinical setting (hospital vs. NHMF), geography (including proximity to the major urban center, Phnom Penh), and poverty level. The results build and expand on previously published data on pandemic health resource distribution in Cambodia [12,17] by focusing the analysis at the OD rather than Province level, allowing better appreciation of varying patterns in resource distribution. Additionally, the introduction of a measure of socioeconomic status, the PPR, allows for novel analysis of resource distribution by poverty quintile, an important consideration given the higher vulnerability of poor communities to pandemic influenza.

In the event of a pandemic, inpatient beds can be redistributed to affected areas with insufficient resources relatively quickly, particularly among ODs within a given Province. Indeed, the Cambodian Ministry of Health anticipates the need for redistribution of certain resources, such as isolation beds and personal protective equipment (PPE), from areas of high concentration to areas experiencing a pandemic-related surge in demand. Detailed knowledge of existing resource density by OD is, thus, essential for rapid and efficient redistribution in the event of a pandemic.

In terms of doctors and nurses, there may be benefit to temporarily deploying medical personnel most heavily concentrated in Phnom Penh to remote ODs in the event of a pandemic influenza, assuming that affected individuals are unable to voluntarily travel to the capital to seek care (either due to distance or illness severity). The fact that the distribution of doctor and nurses by OD exhibited markedly different patterns, particularly by PPR, is worth noting and raises the question of whether enhancing clinical capacity of nurses to serve as physician extenders in areas of the country where doctors are scarce might also be beneficial in a resource-constrained setting. Indeed, this may present an opportunity to improve pandemic preparedness and prevention through maximizing population health, as nurses represent core workforce capacity for basic primary care provision and many public health education activities. At the same time, increased investment in health care workers (both doctors and nurses) in relatively undersupplied ODs is prudent. In order to achieve its articulated goals of improving baseline health in three identified priority areas – reducing newborn, child and maternal morbidity and mortality while improving reproductive health; reducing morbidity and mortality attributable to communicable diseases, including HIV/AIDS, tuberculosis, and malaria; and reducing the burden of non-communicable and other diseases – the Ministry of Health has acknowledged the importance of improving health service delivery at the OD level, including through more effective human resource planning and management [14,25]. Focusing future government health spending on incentivizing health care worker deployment in underserved areas and providing the necessary durable medical equipment to support such an influx in workers would not only strengthen future pandemic mitigation strategies but would aid the country in achieving the goals of the Health Sector Strategic Plan 2008–2015 (HSP) as well. Indeed, health service delivery and human resources for health are two of five strategic areas identified in the HSP, and the Cambodian Ministry of Health has explicitly linked successful implementation of this plan with more equitable access and health outcomes for all Cambodians [25].

The availability of oseltamivir reported here must be interpreted within the regional context in which Cambodia is embedded. As a member of ASEAN since 1999, Cambodia received an initial national stockpile of antivirals (as well as PPE) from the Japan-ASEAN Antiviral Stockpile for ASEAN Countries and is eligible to draw on additional stores from the central stockpile storage site in Singapore in the event of a pandemic. Cambodia was the site of a test exercise in early 2007 to determine adequacy of protocols for redistributing antivirals and PPE from the central stockpile, suggesting that such a strategy could efficiently augment Cambodia’s existing supplies of these important resources [26].

For resources that require the presence of trained medical personnel and reliable electricity to be clinically useful, such as ventilators, it will likely prove most efficient to refer or transport patients to areas where these resources are known to be concentrated. Given the low absolute numbers of ventilators available in Cambodia and the logistical challenges (i.e. requirements for attendant electricity and personnel) involved in mobilizing ventilators either domestically or from other countries in the region, it is likely that the capacity of even the most well-supplied areas would be rapidly overwhelmed during a pandemic by patients seeking care at referral hospitals.

It is important to emphasize that the data presented here reflect only the distribution of selected resources provided through the public health care system in Cambodia and do not account for complementary resources available in the private and non-government organization (NGO) sectors. Given the historical development of the Cambodian health care system over the past few decades, with substantial reliance on donor investment and NGO health programming activities in addition to an intentional strategy of decentralized health system management and public-private partnerships, a comprehensive assessment of resource distribution requires complementary data on resources available in these other sectors. Indeed, an estimated 50-60% of all health treatments are provided by the private sector and 15-20% by practitioners outside the traditional medical sector (i.e. by drug vendors, religious leaders, and traditional healers), compared with approximately 20% through government facilities [27,28]. Thus, it may be appropriate, for instance, for the Ministry of Health to focus public health resources in areas where private health care services and NGO-supported services have traditionally been absent. Additionally, this analysis does not include any measures of health resource accessibility or health outcomes, as the data required for such an analysis were beyond the scope of this study. For these reasons, we cannot draw conclusions about the appropriateness or equity of resource distribution from these data; indeed, it may be fully intentional (and clinically reasonable) to concentrate health care workers in areas of high population density and numerous referral hospitals, such as Phnom Penh, and equally logical to maximize the utility of scarce resources such as ventilators by locating them in facilities where they can be reliably maintained and accessed.

## Conclusions

There is substantial variation in health care resources important for pandemic influenza response across Cambodia. This diversity is related to a number of factors, including geographic location, resource type, clinical setting, and poverty rates at the OD level. Absolute availability of specific key resources also plays a role, as resources that are scarce nationwide are concentrated in selected areas. The implications of these findings vary by resource type, as the strategies for distribution and improved accessibility must take into account clinical appropriateness as well as resources available from outside the country. Further assessment of the feasibility and appropriateness of resource distribution options requires a more comprehensive understanding of complementary health resources available in the private and NGO sectors. In conjunction with such data, the resource distributions presented here will prove useful for strategic health resource investment around the country, both for future pandemic mitigation and to strengthen the baseline functioning of the Cambodian health care system.

Understanding the existing distribution of selected healthcare resources important for treating severe influenza is a necessary condition for ensuring equity in future pandemic mitigation planning in Cambodia. Health-related equity is a broad concept that encompasses health service financing, health service delivery, and health outcomes. Although there are numerous definitions for health equity, most authors agree that achieving equity in health requires minimizing avoidable inequities (i.e. disparities between groups of people as defined by gender, race or ethnicity, socioeconomic status, or geographic location) in health determinants and outcomes by ensuring equal access to a minimum standard of healthcare [15,29-31]. The conditions that affect access are numerous, and include availability of effective health services (including infrastructure, staff, medications, equipment) and funding mechanisms [15]. As there were insufficient available data to link the availability of inpatient beds, doctors, nurses, ventilators and oseltamivir to patient access patterns or health outcomes, we cannot make any claims regarding the equity of the distributions reported here. However, as resource availability is an essential component of health service delivery, we believe this analysis represents an important contribution toward future equity analyses of pandemic mitigation planning in Cambodia. Specifically, it draws attention to differences in selected resource distribution across the country and thus lays the foundation for further study of potential inequities by socioeconomic status and geographic location. Given the disproportionately high impact of pandemic influenza on the health of poor populations, further exploration of vertical equity (i.e. differential access for unequal need) in pandemic mitigation planning in Cambodia is of vital importance.

## Abbreviations

CDB: Commune Database; DHO: District Health Office; GIS: Geographical information system; GIZ: Deutsche Gesellschaft für Internationale Zusammenarbeit; HSP: Health Sector Strategic Plan 2008–2015; LISA: Local indicators of spatial autocorrelation; NCDD: Cambodian National Committee for Sub-National Democratic Development; NGO: Non-governmental organization; NHMF: Non-hospital medical facilities; OD: Operational District; PPE: Personal protective equipment; PPR: Predicted Family Poverty Rate.

## Competing interests

SUSK: The author declares no competing interests.

JWR: JWR has worked on an unrelated project funded by Hoffman La Roche, the maker of oseltamivir.

TD: The author declares no competing interests.

IC: The author declares no competing interests.

KB: The author declares no competing interests.

ST: The author declares no competing interests.

RC: RC has held grants unrelated to this research from Hoffman La-Roche, the maker of oseltamivir.

## Authors’ contributions

SSK carried out the socioeconomic analysis and drafted the manuscript. JWR participated in study coordination and data analysis and helped draft the manuscript. TD coordinated data analysis and helped draft the manuscript. IC performed the GIS analysis and drafted corresponding sections of the manuscript. KB and ST facilitated data collection and participated in resource characterization. RC conceived, designed and coordinated the study. All authors read and approved the final manuscript.

## Supplementary Material

Additional file 1 **Map of Operational Districts (ODs) in Cambodia.** Description: Map enumerating the Operational Districts (ODs) in Cambodia with corresponding OD names, for reference in interpreting Figures 3, 4, 5, 6. Click here for file

Additional file 2 **Lorenz Curves of Healthcare Resource Densities (Province and OD).** Description: Lorenz curves showing inequality in healthcare resource densities per capita across Provinces and Operational Districts (ODs) in Cambodia. Click here for file

Additional file 3 **NHMF-based Doctors per Capita, Excluding Phnom Penh (OD).** Description: Per capita density of doctors based in non-hospital medical facilities stratified by Predicted Family Poverty Rate at the Operational District level, excluding Phnom Penh. Click here for file

Additional file 4 **Oseltamivir and Ventilators per Capita (OD).** Description: Per capita density of oseltamivir and ventilators (both adult and pediatric) stratified by Predicted Family Poverty Rate at the Operational District level. Click here for file
