# Supplementary figures and images for: Distribution of selected healthcare resources for influenza pandemic response in Cambodia
Source: Int J Equity Health. 2013 Oct 4;12:82. doi: 10.1186/1475-9276-12-82 (PMC3851316; doi:10.1186/1475-9276-12-82)

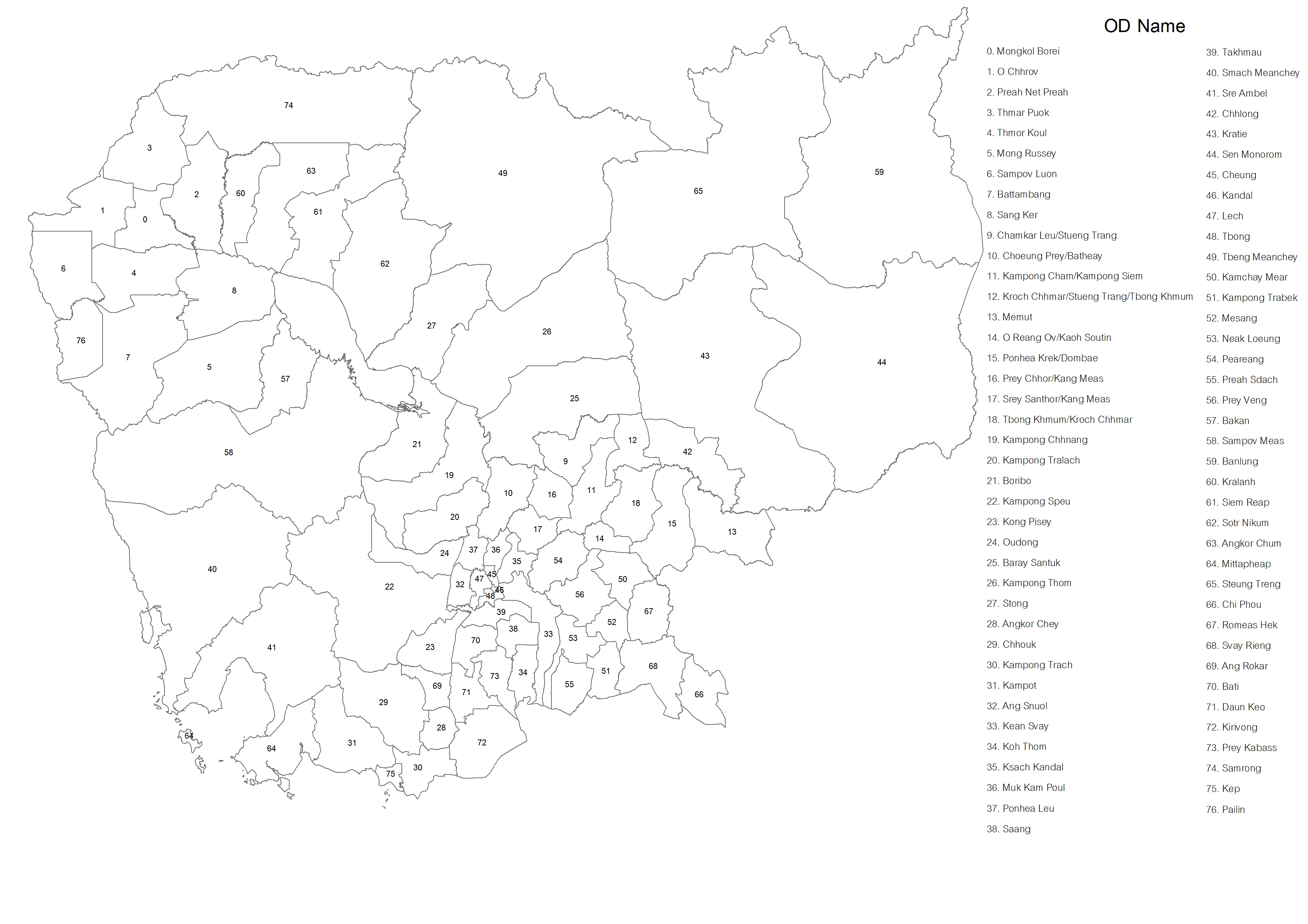

Supplement: Additional file 1 — Map of Operational Districts (ODs) in Cambodia. Description: Map enumerating the Operational Districts (ODs) in Cambodia with corresponding OD names, for reference in interpreting Figures 3, 4, 5, 6. [file 1475-9276-12-82-S1.jpeg]

**Province level**

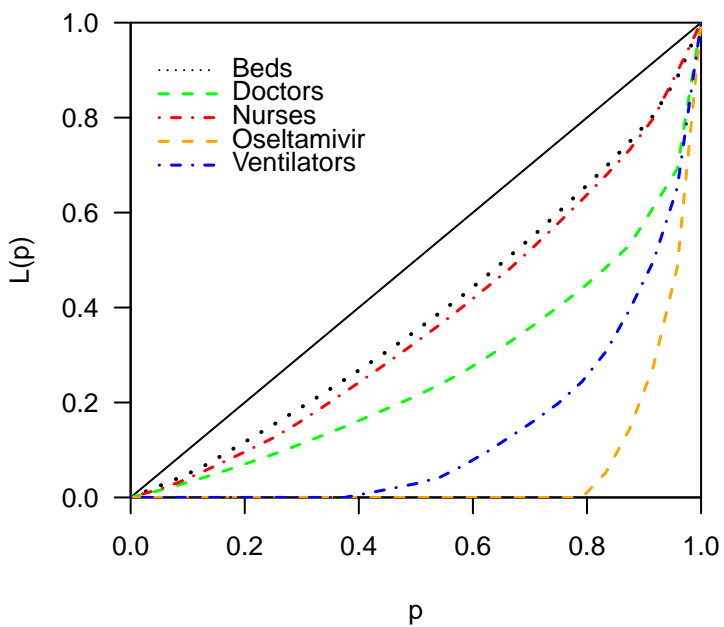

**Province level  
(population weighted)**

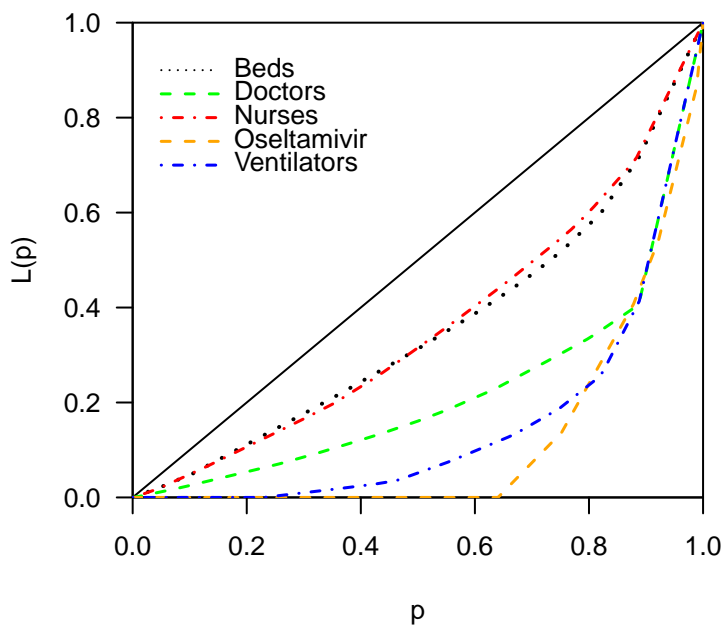

**OD level**

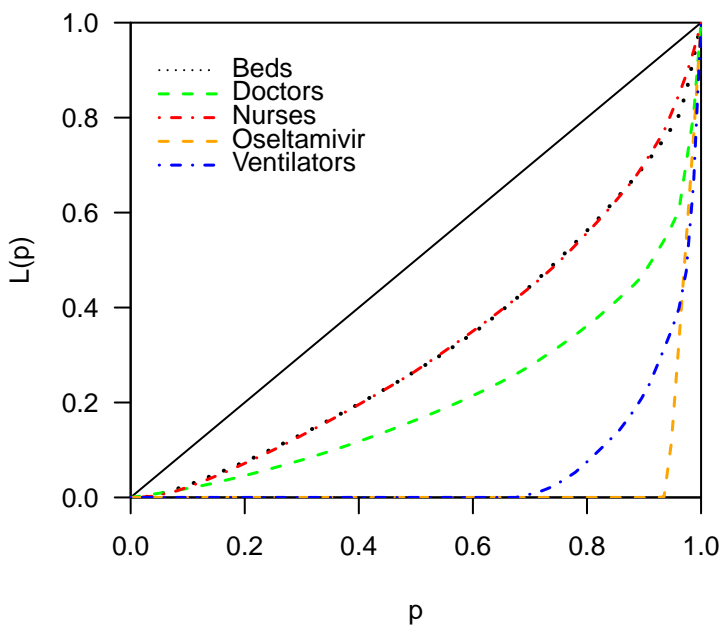

**OD level  
(population weighted)**

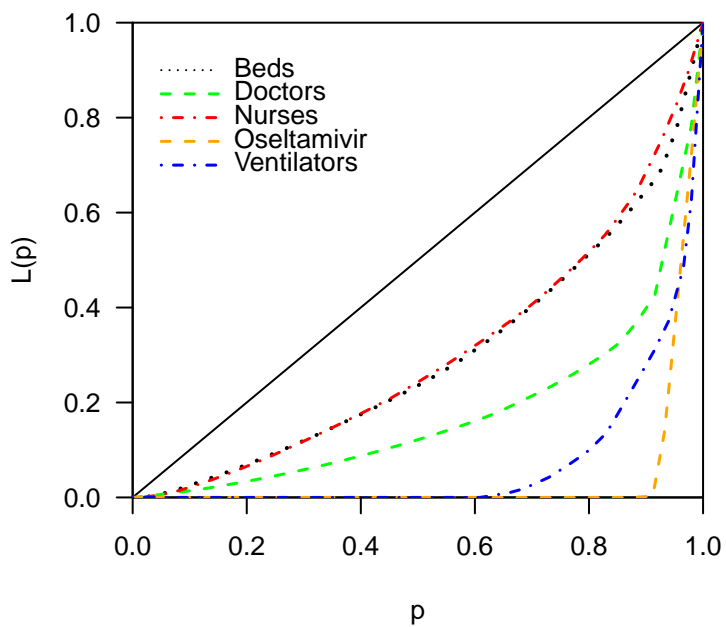

Supplement: Additional file 2 — Lorenz Curves of Healthcare Resource Densities (Province and OD). Description: Lorenz curves showing inequality in healthcare resource densities per capita across Provinces and Operational Districts (ODs) in Cambodia. [file 1475-9276-12-82-S2.pdf]

**NHMF-based Doctors per Capita, Excluding Phnom Penh**

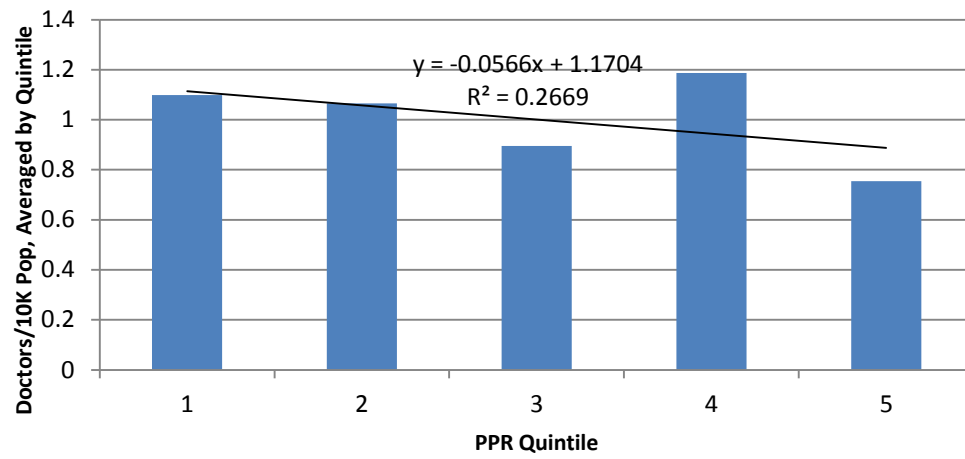

Supplement: Additional file 3 — NHMF-based Doctors per Capita, Excluding Phnom Penh (OD). Description: Per capita density of doctors based in non-hospital medical facilities stratified by Predicted Family Poverty Rate at the Operational District level, excluding Phnom Penh. [file 1475-9276-12-82-S3.pdf]

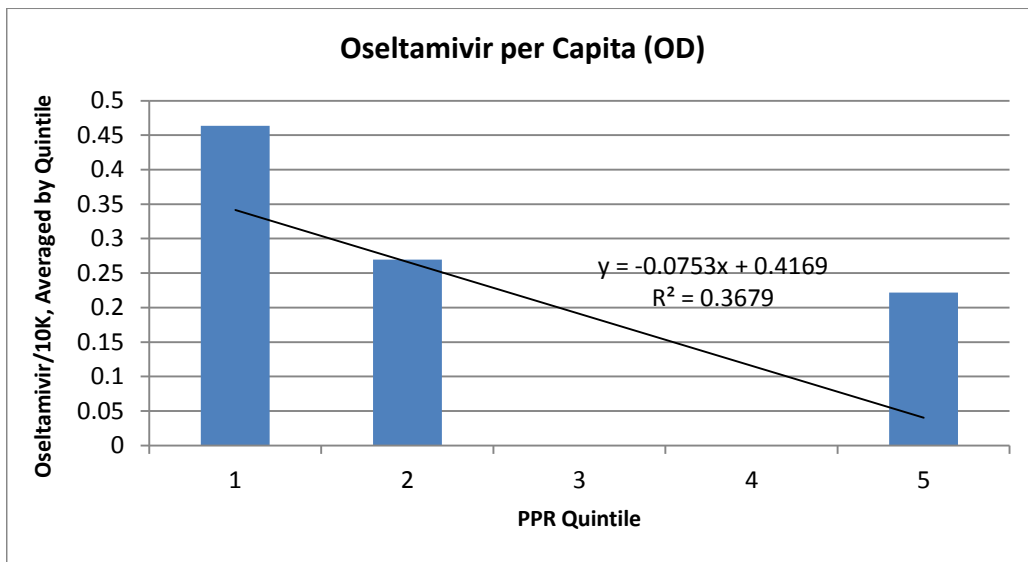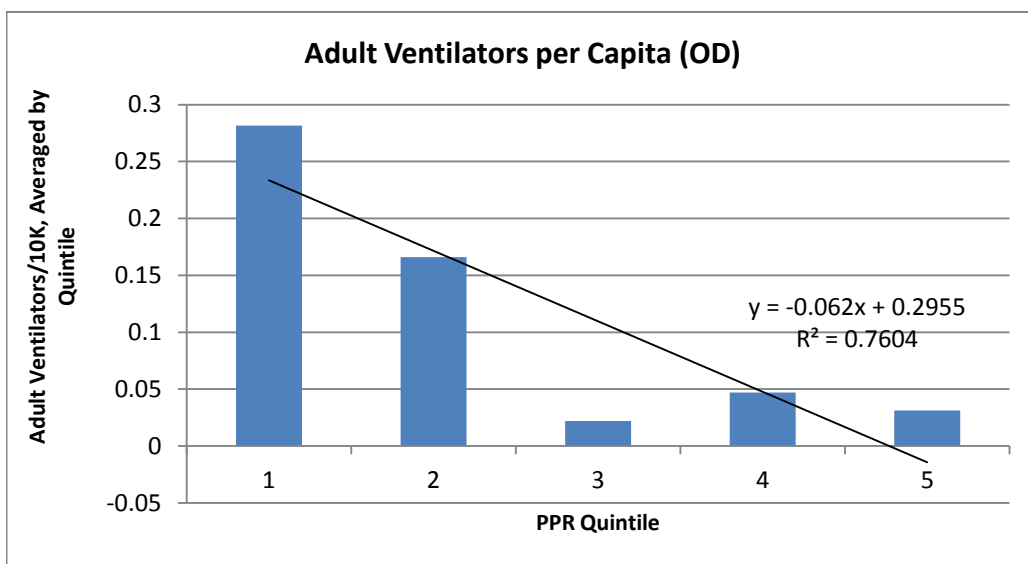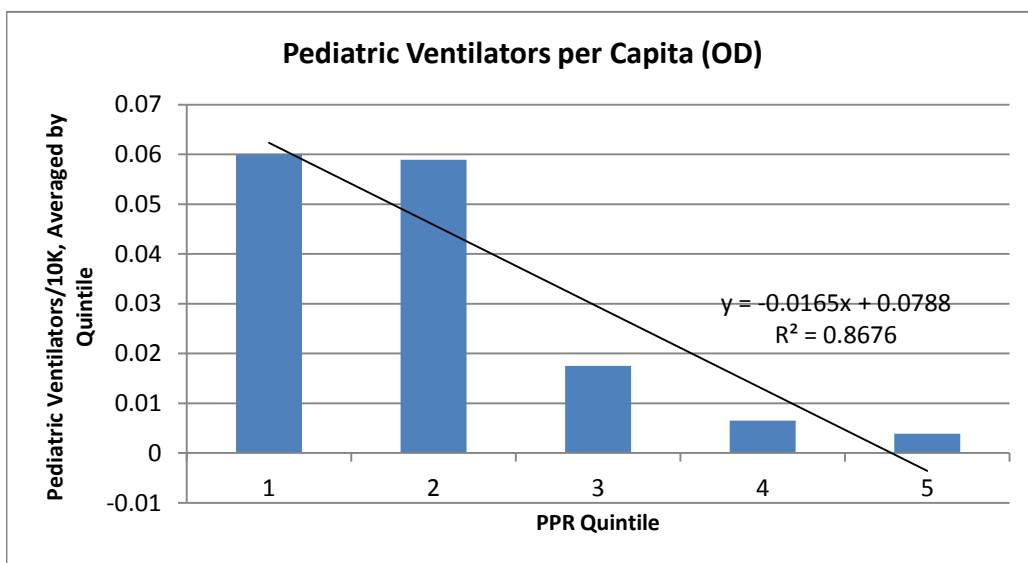

Supplement: Additional file 4 — Oseltamivir and Ventilators per Capita (OD). Description: Per capita density of oseltamivir and ventilators (both adult and pediatric) stratified by Predicted Family Poverty Rate at the Operational District level. [file 1475-9276-12-82-S4.pdf]
